# Lacrimal Hypofunction as a New Mechanism of Dry Eye in Visual Display Terminal Users

**DOI:** 10.1371/journal.pone.0011119

**Published:** 2010-06-15

**Authors:** Shigeru Nakamura, Shigeru Kinoshita, Norihiko Yokoi, Yoko Ogawa, Michiko Shibuya, Hideo Nakashima, Ryuji Hisamura, Toshihiro Imada, Tomohiro Imagawa, Masato Uehara, Izumi Shibuya, Murat Dogru, Samantha Ward, Kazuo Tsubota

**Affiliations:** 1 Department of Ophthalmology, Keio University School of Medicine, Tokyo, Japan; 2 Research Center, Ophtecs Corporation, Toyooka, Japan; 3 Department of Ophthalmology, Kyoto Prefectural University of Medicine, Kyoto, Japan; 4 Department of Veterinary Anatomy, Faculty of Agriculture, Tottori University, Tottori, Japan; 5 Department of Veterinary Physiology, Faculty of Agriculture, Tottori University, Tottori, Japan; 6 Johnson & Johnson Ocular Surface Visual Optics Department, Keio University School of Medicine, Tokyo, Japan; Johns Hopkins University, United States of America

## Abstract

**Background:**

Dry eye has shown a marked increase due to visual display terminal (VDT) use. It remains unclear whether reduced blinking while focusing can have a direct deleterious impact on the lacrimal gland function. To address this issue that potentially affects the life quality, we conducted a large-scale epidemiological study of VDT users and an animal study.

**Methodology/Principal Findings:**

Cross sectional survey carried out in Japan. A total of 1025 office workers who use VDT were enrolled. The association between VDT work duration and changes in tear film status, precorneal tear stability, lipid layer status and tear secretion were analyzed. For the animal model study, the rat VDT user model, placing rats onto a balance swing in combination with exposure to an evaporative environment was used to analyze lacrimal gland function. There was no positive relationship between VDT working duration and change in tear film stability and lipid layer status. The odds ratio for decrease in Schirmer score, index of tear secretion, were significantly increased with VDT working year (*P* = 0.012) and time (*P* = 0.005). The rat VDT user model, showed chronic reduction of tear secretion and was accompanied by an impairment of the lacrimal gland function and morphology. This dysfunction was recovered when rats were moved to resting conditions without the swing.

**Conclusions/Significance:**

These data suggest that lacrimal gland hypofunction is associated with VDT use and may be a critical mechanism for VDT-associated dry eye. We believe this to be the first mechanistic link to the pathogenesis of dry eye in office workers.

## Introduction

Working related to video display terminals (VDT) has been increasing in the office workplace and their use is growing because of the rapid advance of information technology. Intel estimates that there are close to one billion Internet-connected personal computers throughout the world [1]. People regularly using a VDT have demonstrated a higher incidence of musculoskeletal disorders, eyestrain, and dry eyes [2], [3]. Dry eye has shown a marked increase due to VDT use, and has become a significant health issue affecting the quality of life in industrialized countries [4]. Dry eye syndrome is defined as a disturbance in tear film physiology that leads to various abnormal states of ocular surface cells that elevate the incidence of ocular surface disorders and infection [5]. Research has also proved that, although dry eye syndrome is not a frequent cause of blindness such as retinal diseases, abnormal functional visual acuity is a major symptom of dry eye [6], [7].

**Figure 1:**
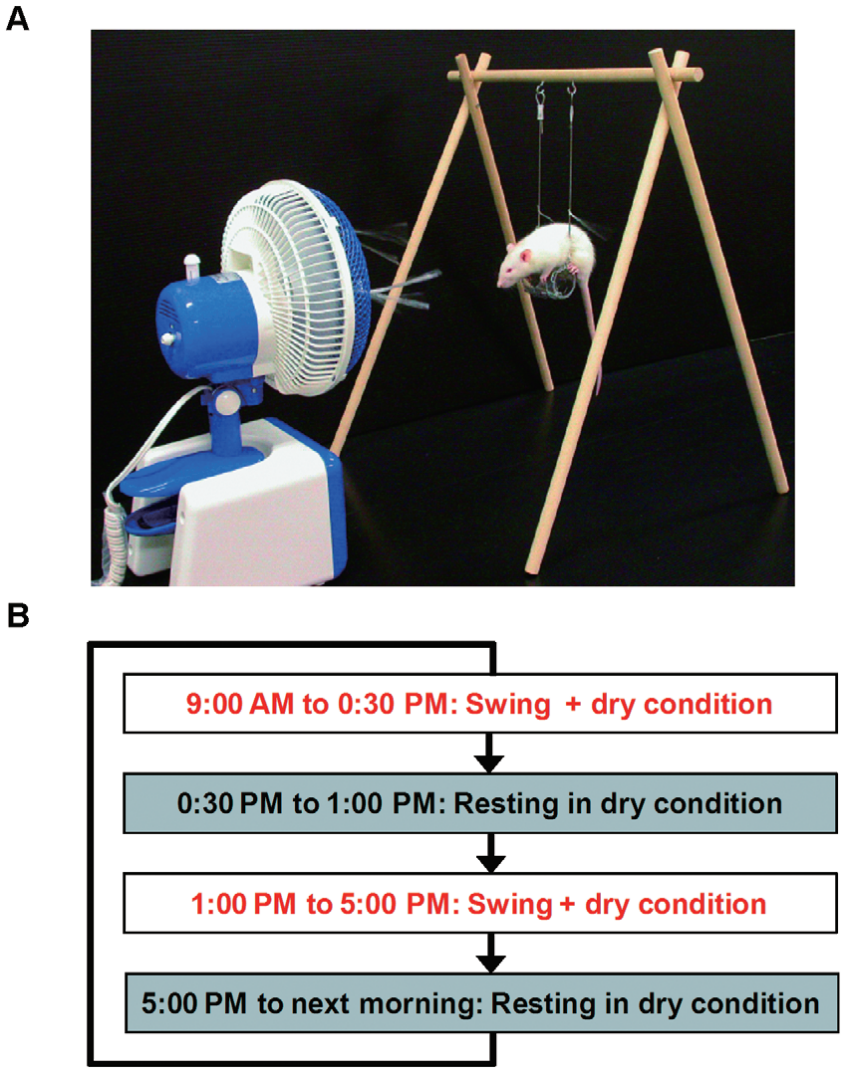
Rat VDT user model. (A) Image of rat VDT user model. (B) Schematic representation of daily experimental schedule for rat VDT user model. This series of treatments was repeated for up to 20 days.

The tear film covering the cornea and conjunctiva consists of lipid, aqueous and mucin layers [8]. Each component of the tear film is essential for maintaining a properly controlled environment for the ocular surface. There are two main causes of dry eye syndrome: Tear-deficient dry eye and evaporative dry eye [9]. Tear-deficient dry eye is characterized primarily by a lack of tear secretion by the lacrimal glands. Evaporative dry eye is characterized by excessive evaporative loss of tears from the ocular surface that leads to tear film instability with normal tear secretion. Excess evaporation of tear fluid due to reduced blinking while focusing has been considered to be a major causative factor in VDT-associated dry eye [10], [11]. However, there has been no proposed mechanism that accounts for progressive worsening of dry eye in VDT users, which remains an important unexplored quality of life issue. The mechanism for this has yet to be fully understood due to the lack of information obtained from a large-scale epidemiological study of VDT users and an appropriate animal model for dry eye. Animal models have not been developed because the etiology of computer work associated with health problems is complicated. Furthermore, no way has been found to reduce the blink frequency of animals and because animals do not use computers. To mimic VDT use which is characterized by lack of blinking, low humidity occupational environment, and sustained static postures during repetitive tasks, we have created a novel rat model. The procedure based on the concept that gazing is not necessarily only observed in the concentrated tasks such as VDT use, but also can be observed in the spatial orientation that is required for the maintenance of posture, similar to that seen in tightrope walkers [12]. Using our novel procedure, we were able to simulate the stressful conditions of VDT use. The procedure involved placing rats on a swing in combination with exposure to an evaporative environment. In this study, the animal model reveals the blink frequency being reduced to one-third of the non-swing riding levels, which is similar to the results that have been reported by VDT users [13] (Figure 1A, Movie S1, S2, S3). In the present study we characterized the etiology of VDT-associated dry eye by epidemiological study of VDT users and investigated the underlying mechanism and precautions by a rat model that mimics the condition of VDT users.

## Results

### Decreased tear secretion in VDT users depended upon on the number of years and daily hourly usage

The characteristics of the study population are shown in Table 1.

**Table 1:** Characteristics of study population.

Variable	Estimate*
Age (years)	35.6±10.1
Gender (male) (%)	335/601 (56)
VDT working year (years)	8.2±5.7
Daily VDT hour (hours)	5.1±2.7
BUT (sec)	5.7±2.7
DR-1 grade	2.3±0.5
Schirmer score (mm/min)	19.7±10.2

*Values represent mean ± standard deviation.

There was no positive relationship between duration of VDT working year and daily using time and change in tear film break up time (BUT) (Table 2) and DR-1 (Table 3) values. In contrast, the odds ratio (OR) for decrease in Schirmer score were significantly increased 8–12 (OR = 2.49, 95% confidence interval [CI]: 1.02–6.55) and more than 12 years (OR = 3.61, 95% CI: 1.39–10.26) compared with less than 4 years, and more than 8 hours (OR = 4.27, 95% CI: 1.47–13.66) of daily VDT working time compared with less than 2 hours. Statistically significant dose-response relations were observed for VDT working year (*P* = 0.012) and time (*P* = 0.005) (Table 4). We also performed an analysis of values that examined subjects based on time, i.e., we examined a group that included subjects that had been working for 12 years or more and another group that focused on subjects that worked 8 hours or more per day. No differences for tear film BUT and DR-1 values were found between each working group. Statistically significant reduction in Schirmer test values were found for among these groups (Table S1).

**Table 2:** Logistic regression models of the relationship between decrease in precorneal tear stability in VDT users and working duration.

Category	Sub category	Decrease in tear stability (BUT ≤5 sec)		OR† *	95% C I§
		Cases	Controls		
		(n = 297)	(n = 304)		
**VDT working year**	**<4**	92	86	reference	
	**4–8**	64	66	0.97	0.60, 1.56
	**8–12**	80	86	0.92	0.59, 1.46
	**≥12**	61	66	0.95	0.56, 1.62
*P*_trend_ = 0.956					
**Daily VDT hour**	**≤2**	50	52	reference	
	**2–4**	80	78	1.07	0.64, 1.79
	**4–6**	64	75	0.89	0.52, 1.50
	**6–8**	82	59	1.50	0.88, 2.57
	**>8**	21	40	0.65	0.32, 1.27
*P*_trend_ = 0.866					

†OR: odds ratio;

*Adjusted for gender and age;

§Confidence interval.

**Table 3:** Logistic regression analysis of the relationship between tear lipid layer status in VDT users and working duration.

Category	Sub category	Tear lipid layer status (DR-1 grade >3)		OR† *	95% C I§
		Cases	Controls		
		(n = 109)	(n = 492)		
**VDT working year**	**<4**	36	142	reference	
	**4–8**	22	108	0.90	0.49, 1.64
	**8–12**	29	137	0.99	0.55, 1.76
	**≥12**	22	105	1.05	0.53, 2.09
*P*_trend_ = 0.896					
**Daily VDT hour**	**≤2**	20	82	reference	
	**2–4**	25	133	0.75	0.39, 1.45
	**4–6**	20	119	0.66	0.33, 1.32
	**6–8**	24	117	0.80	0.41, 1.59
	**>8**	20	41	2.03	0.96, 4.34
*P*_trend_ = 0.190					

†OR: odds ratio;

*Adjusted for gender and age;

§Confidence interval.

**Table 4:** Logistic regression analysis of the relationship between decrease in tear secretion in VDT users and working duration.

Category	Sub category	Decrease in tear secretion (Schirmer score ≤5 mm)		OR† *	95% C I§
		Cases	Controls		
		(n = 57)	(n = 544)		
**VDT working year**	**<4**	8	170	reference	
	**4–8**	11	119	1.84	0.69, 5.04
	**8–12**	18	148	2.49	1.02, 6.55
	**≥12**	20	107	3.61	1.39, 10.26
*P*_trend_ = 0.012					
**Daily VDT hour**	**≤2**	6	96	reference	
	**2–4**	13	145	1.47	0.55, 4.39
	**4–6**	10	129	1.35	0.47, 4.23
	**6–8**	16	125	2.30	0.87, 6.89
	**>8**	12	49	4.27	1.47, 13.66
*P*_trend_ = 0.005					

†OR: odds ratio;

*Adjusted for gender and age;

§Confidence interval.

### Rat VDT user model

**Figure 2:**
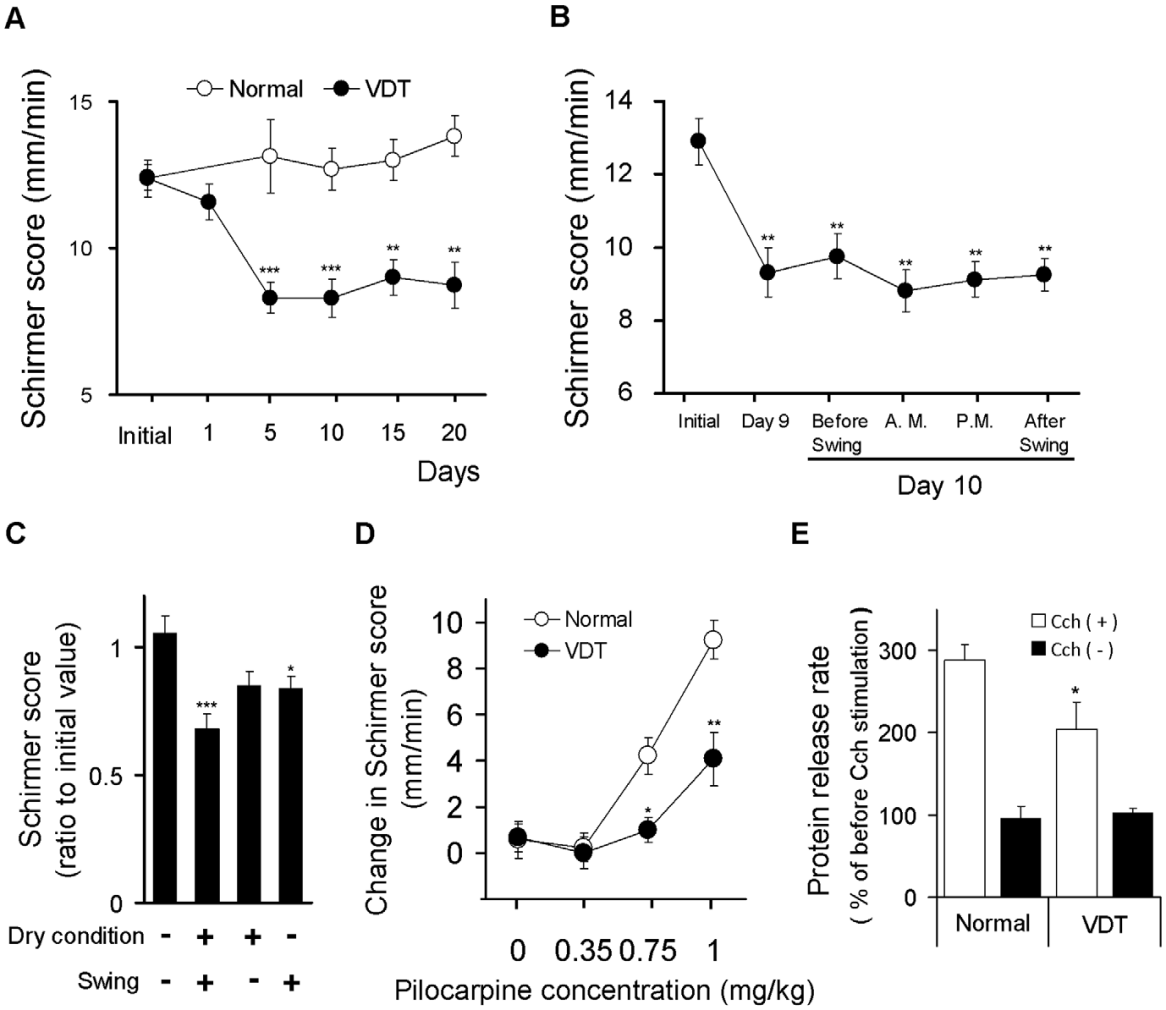
Lacrimal function is impaired in rat VDT user model. (A) Changes in tear secretion during 20 days of swing use. For rats repeating the daily experimental cycle, the Schirmer test was performed on days 1, 5, 10, 15 and 20. Data represent the mean ± SEM for 16 eyes. ** *P*<0.01, *** *P*<0.001 versus normal. (B) Circadian variation of the tear production in rats placed in the swing. Changes in the Schirmer score were evaluated on days 9 and 10. All data represent the mean ± SEM for 16 eyes. ** *P*<0.01 versus initial value. (C) Effect of stressed conditions on the Schirmer score. Changes of tear secretion were measured 10 days after treatment with or with-out swing or dry condition. Data present the mean ± SEM for 16 eyes. * *P*<0.05, *** *P*<0.001 versus the normal condition group. (D) Increase in tear secretion by systemic parasympathetic stimulation. Tear fluid secretion was stimulated by subcutaneous injection of pilocarpine hydrochloride on day 10. Increase in tear secretion was calculated by subtraction of the Schirmer value from before pilocarpine injection. Data represent the mean ± SEM for 10 to 23 eyes. * *P*<0.05, ** *P*<0.01 versus the normal. (E) Changes in protein secretion capacity in LG by parasympathetic stimulation. Changes in the protein release after stimulation by carbachol (Cch) using isolated LG on day 10. The protein secretion rate was calculated as a percentage of before Cch stimulation. Data represent the mean ± SEM for 16 LG. * *P*<0.05 versus the normal with Cch stimulation.

In our previous study, we demonstrated that a rat under the swing treatment induced a decrease in blink frequency, the appearance of corneal surface disorder and a decrease in tear secretion [14]. First, to further confirm tear secretion is chronically reduced in this model, we repeated treatments corresponding to the work-rest-sleep cycle of office workers on a daily basis up to 20 days (Figure 1B). Significant reduction of tear secretion was sustained from 5 days to 20 days compared to age-matched normal rat (Figure 2A). We selected 10 days of treatment for further examinations, because the reduction in tear secretion had plateaued up to this time point. To confirm tear secretion was reduced even when the rats were not on the swing, circadian variation of tear secretion was examined. On day 10, we performed the Schirmer test 4 times: before, two times during (10 AM and 1 PM) and after swing placement. A significant decrease appeared compared to the initial value at all time points and few changes were observed among all time points measured (Figure 2B). Next, we investigated whether reduction of tear secretion is specifically induced by swing treatment. We compared Schirmer scores among three groups: 1) rat placed for 10 days under dry conditions with daily swing placement, 2) dry conditions without swing placement and 3) the standard condition with daily swing placement. The significant reduction of tear secretion was only observed with the swing group. The maximum reduction was observed in swing treatment under the dry condition group. These results suggest that placement on the swing plays a critical role in the reduction of tear secretion in our model, and indicates that the dry condition plays a possible role in the acceleration of this reduction (Figure 2C).

### Tear secretion capacity is impaired in rat VDT user model

Tears are secreted from the LGs and are under the control of the parasympathetic nervous system [15]. To determine that the tear secretion capacity was suppressed during the experimental cycle, we measured changes in tear production after stimulation by a parasympathetic agonist pilocarpine. Dose dependent increase in aqueous tear secretion was observed in normal rats and in the same model after injection of pilocarpine. Consistent with the non-stimulated state, increased aqueous tear production was significantly lowered in this model compared to the normal rats (Figure 2D). Tear proteins are major components of tears as well as the aqueous phase [16]. To evaluate the changes of the LG protein secretion capacity in the swing group, we measured protein secretion from the isolated LG in vitro. The effect of carbachol (Cch), a cholinergic stimulant, was significantly reduced in the LG of the VDT user model as compared to that of untreated rats (Figure 2E).

### Rat VDT user model causes alterations in lacrimal gland morphology

**Figure 3:**
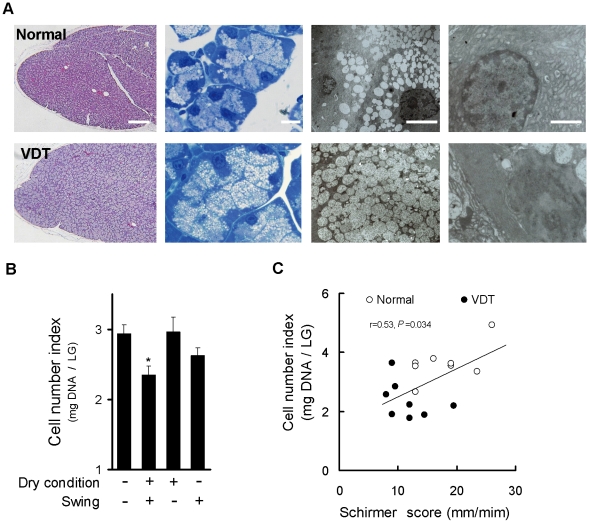
Rat VDT user model causes alterations in lacrimal gland morphology. (A) Left: H & E staining. Left center: Toluidine blue staining. Right center and right: Electron microscopic analysis of acinar cells. Images showing expanded aciner cells accompanied by accumulated enlarged secretory vesicle in the cytoplasm (center), decresed endoplasmic reticulum and increase in the nuclei with dark neucleoplasm (Right) of LG on day 10. Scale bars: Left  = 200 µm; Left and right center  = 10 µm; Right  = 4 µm. (B) Changes in total cell number of LG. Changes of LG cell number were measured 10 days after treatment with or without swing or dry condition. Quantification of LG number was calculated by deoxyribonucleic acid content of the LG. Data represent the mean ± SEM for 8 to 16 eyes. * *P*<0.05 versus the without swing and dry condition. (C) Correlation between tear production and LG cell number. Pearsons correlation coefficient testing was r = 0.53 (*P* = 0.034, n = 16).

In the rat VDT user model, dramatic histopathological alterations occurred. The entire area of the LG was occupied by enlarged acini with expanded cytoplasm compared to the normal group (Figure 3A left). Quantitative analysis showed that total cell was decreased (Figure 3B). Consistent with changes in tear production, there were morphological changes that specifically developed during our experimental cycle. A high correlation was established between tear production and acinar cell number (Figure 3C). To further characterize these changes, we assessed the fine structure of the acinar component by toluidine blue staining and electron microscopy. In the expanded acinar cells found in the LG of the VDT user model, the cytoplasm was filled with accumulated enlarged secretory vesicles (Figure 3A left and right center). The organelles, especially in the endoplasmic reticulum were apparently decreased, and the nuclei with dark neucleoplasm were increased in the LG acinar cells, which characterizes damage of cellular activity (Figure 3A right).

We have recently experienced one patient who has been using a VDT for more than 12 hours per day for 15 years. Although this patient does not have Sjogren's syndrome, the lacrimal function was severely suppressed and he was treated by lacrimal punctum plug. Similar to our rat model, the lacrimal biopsy showed a greater amount of accumulated secretory vesicles, as compared to normal subjects [17] (Figure S1, Materials and Methods S1).

### Modifying daily treatment schedule restores tear secretion capacity

**Figure 4:**
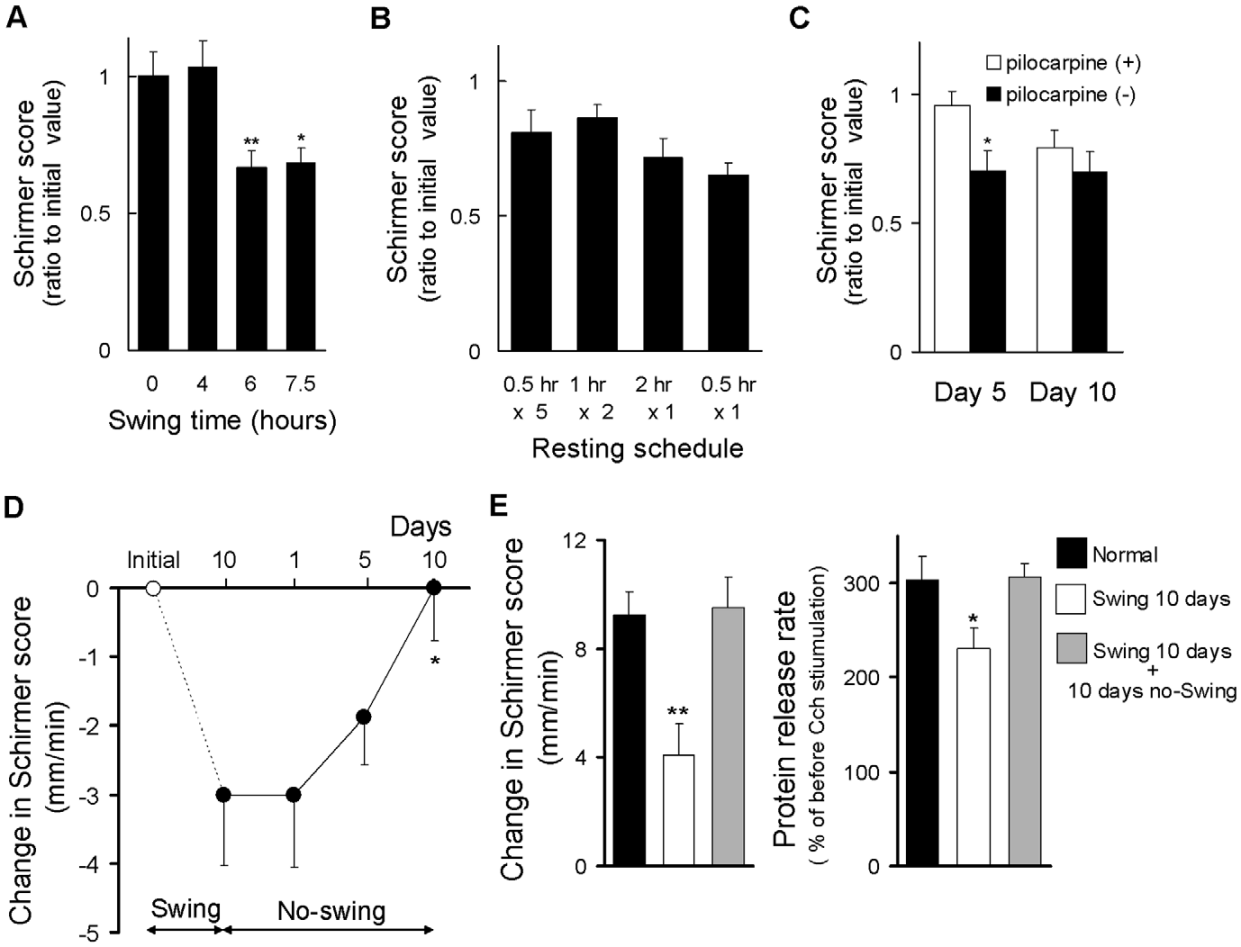
Recovery of tear secretion with long-term rest without swing activity. (A) Effect of shortening the time spent on the swing. Ratios to initial value were calculated. Data represent the mean ± SEM for 16 eyes. * *P*<0.05, ** *P*<0.01 versus 0 hour riding swing group. (B) Effects of changing resting patterns in tear secretion. Ratios to initial value were calculated. Data represent the mean ± SEM for 16 eyes. (C) Effect of stimulation on the lacrimal function for the two groups, with and without pilocarpine injection. Ratios to initial values were calculated. Data represent the mean ± SEM for 16 eyes. (D) Effect of extended rest period without the swing. Data represent the mean ± SEM for 8 to 16 eyes. * *P*<0.05 versus day 10. (E) Effect of extended rest period without the swing on tear secretion in response to parasympathetic stimulation (Left) and recovery of protein released from isolated LG (Right). Data represent the mean ± SEM for 8 to 16 eyes. * *P*<0.05 versus the normal.

To address effective treatment for preservation of lacrimal dysfunction in VDT users, we studied the effects of modifying daily treatment schedules of our rat model. In the epidemiological study of office workers, we found that reduction of tear secretion was associated with daily working duration (Table 3). To study the relationship between rats and humans, shortening the time spent on the swing was performed. Significant reduction of tear secretion was diminished when the time spent on the swing was shortened to less than 4 hours (Figure 4A). Short, frequent breaks are recommended to relieve visual stress due to VDT use. To test this, we modified the resting pattern in our rat model. Even with increased frequency or continuous resting time from the swing, there was only a slight restoration of the lacrimal function (Figure 4B). We next investigated the effect of stimulation of the lacrimal function by systemic injection of a muscarinic cholinergic agonist pilocarpine. Pilocarpine has been proven to be efficacious for the treatment of dry mouth and dry eye associated with Sjogren's Syndrome [18], [19]. After 5 days of pilocarpine stimulation after riding the swing, a significant improvement of tear secretion was observed. However, on day 10, the effect of relief was diminished to the point where it was almost equal to the non-stimulated level (Figure 4C). A certain period of resting without using computer is a conclusive way to relieve VDT stress. When rats were placed under resting conditions after 10 days of swing placement, the decreased tear secretion gradually recovered and by day 10 had almost returned to its initial value (Figure 4D). This recovery was also noted for stimulated tear secretion (Figure 4E left), protein secretion capacity (Figure 4E right), cell number and morphology (Figure S2A). Corresponding to the Schirmer value, corneal epithelial disorder, a characteristic feature of dry eye, was gradually recovered and returned to almost the initial value at 10 days of the resting condition (Figure S2B, Materials and Methods S1).

## Discussion

Although VDT use is defined as a cause of dry eye, little is known about the relationship between tear film status and VDT use. It has been hypothesized that excess evaporation of tear fluid due to extended blinking interval while gazing is a causative factor in VDT-associated dry eye [10], [11]. Contrary to expectation, in the present study, there was no relationship between duration of VDT work and decrease in precorneal tear stability. In addition, the status of tear film lipid layer, which protects the aqueous tears from evaporating, had no relationship between duration of VDT work. A possible explanation may be that the ophthalmic examinations were not performed under working conditions with the VDT while blinking was reduced. Therefore, this result suggests that excess evaporation may be an exclusive factor related to transient, not chronic, dry eye symptoms during VDT work such as dryness or ocular fatigue.

In the present study, duration of VDT use has an etiologic association with a decrease in tear secretion. These results suggest that chronic reduction of tear production may be induced by VDT use, and indicates that lacrimal hypofunction is the critical mechanism involved in progressive worsening of VDT users dry eye. In order to prevent the occurrence of dry eyes as well as maintain the ocular surface health, attention needs to be paid to the total lifetime working hours.

The mechanism(s) underlying the observed association between VDT and decreased tear secretion are not defined in the present human study [10], [11]. To investigate the pathogenic mechanism of reduction of tear secretion, which appeared among VDT users, we developed an *in vivo* animal model. In our rat VDT user model, persistent strain by riding on the swing in combination with exposure to an evaporative environment showed persistent decrease in tear secretion. In addition, corresponding to the human study, reduction of tear secretion depended on strain duration. These observations establish that this model could be applicable in the investigation of the etiology of dry eye in VDT use.

Tear secretion is primarily under the control of the sensory, sympathetic and parasympathetic nervous systems [15]. Here we show that an increase in tear fluid and protein secretion by parasympathetic stimulation was suppressed in our rat VDT user model. These findings suggest the presence of damage in tear secretion function in our model. Acinar epithelial cells of the LG are the major cell type responsible for the production and regulation of fluid and protein on the ocular surface. These cells exocytose the contents of mature secretory vesicles at their apical membranes in response to secretagogues [20]. In the LG of our rat model, the decrease in acinar cell number was induced. The characteristic features of morphological change in the LG were enlargement of acinar cells accompanied by filling with an increased volume of secretory vesicles and loss of intracellular cell structure. Basal tear secretion is maintained by a continuous neural reflex from the corneal and conjunctival sensory nerves that is associated with blinking [21]. Total interruption of sensory input to the LG by denervation was reported to produce secretory accumulation of secretory granules [22] or loss of the acinar cell structure [23]. In the parotid gland, which has a secretory mechanism analogous to the LG, disuse atrophy is exhibited by reduced reflex stimulations for salivary secretion that is normally generated in response to masticatory activity [24], [25]. In addition, the accumulation of secretory vesicles in acinar cells was observed in the LG [26], salivary gland [27], [28] or pancreas [29] of exocrine-related molecule knock-out mice. These observations seen in the rats on the swing suggest that disuse dysfunctions of the secretory system in the LG arising from suppressed stimulation associated with blinking is the critical mechanism for reduced tear secretion. Further studies, including the analysis of LG acinar cell tear secretion system, would provide useful explanation for the mechanisms involved.

A few measures were carried out to avoid progression or recovery of lacrimal hypofunction using our rat model. A certain level of delay in progression was achieved by modifying the resting pattern or stimulation of lacrimal function. A complete restoration occurred when rats were moved to an extended duration of resting condition. Our findings indicate that improvement can be expected by modifying daily working conditions or life styles.

Several study limitations need to be considered when interpreting our human results. The data are cross-sectional and do not provide for a direction of causality. Our study population consisted of ethnic Japanese which limits generalizability to other populations. We did not survey other visual tasks such as video games or watching television outside the work environment that may affect blinking pattern. Although dates were adjusted by age and gender, dry eye is a multifactorial disease and we did not study all other confounding factors.

In conclusion, our results from the human and rat studies provided the evidence that not only excess evaporation of tear fluid but also hypofunction of the lacrimal gland contributes to the pathogenesis of VDT-associated dry eye. Since normal tear secretion is essential to maintain homeostasis of ocular surface cells, lacrimal hypofunction is important to the ocular surface just as a pathological situation is likely to promote heart failure in systemic circulation. Our study supports the concept that a proper number of blinks are required in order for healthy LG function to occur. Since VDT use suppresses the blink rate, modifications, such as the use of bigger and clearer characters [30], should be considered when trying to increase the blink rate in addition to modifying daily working conditions or lifestyles. The knowledge obtained from this research relates to not only office workers but also anyone using computer monitors, mobile telephones, handheld games, etc. It is our belief that the current study findings will pave the way for further research or discoveries, including nutraceuticals or pharmacological treatment modalities that will be enable the restoration of LG function.

## Materials and Methods

### Epidemiological study of office workers engaged in VDT use

#### Ethics Statement

Investigations were performed according to the Declaration of Helsinki on Biomedical Research Involving Human Subjects. The epidemiological study was approved by Institutional Review Board of Kyoto Prefectural University of Medicine Review Board and after complete description of the study to the participants, written informed consent was obtained.

#### Study population

We carried out a cross sectional study of 1,025 Japanese (male: 542, female: 483) aged 17 to 73 years (average age 36.0 years, SD 10.0 years) who were engaging in VDT work. The VDT worker is characterized according to the occupational health management guideline for VDT workers [31]. The Japanese Society of Preventative Medicine administered dry eye examinations and questionnaires. Data were collected during the period November 2000 - February 2002. Tear film status was assessed by measuring the precorneal tear stability, lipid layer status and tear secretion. Precorneal tear film stability was assessed by measuring the fluorescein tear film break-up time (BUT). BUT is the interval between the last complete blink and the first appearance of a dry spot on precorneal surface tear film. Tear film lipid layer interferometry (DR-1; Kowa Co., Tokyo, Japan) was performed to evaluate the status of the tear lipid layer. DR-1 interferometry observes the specular reflected light from the tear surface. The classification of tear lipid layer patterns was evaluated by semiquantitative dry eye severity grading as previously reported [32]. (Materials and Methods S1) Examination of tear secretion was carried out by the standard Schirmer 1 test. The Schirmer test is a test to measure change in tear production by the observed wetting of a standardized paper strip placed over the inferior eyelid over a given period of time. All ophthalmic examinations were performed by ophthalmologists. Subjects were asked about the starting year/month of VDT use and duration of VDT use hour/day and day/month.

#### Analyses of relationship between amount of VDT use and tear function

The amount of VDT used per year was calculated from starting year/month to consultation day of the study. Total hours of VDT use per month were calculated and the amount of time of VDT use per day was estimated. Average BUT and Schirmer score from both eyes were analyzed. Unconditional logistic regression analysis was used to calculate odds ratios as an estimate of the relative risk and 95 percent confidence intervals for the association between amount of VDT working duration and the risk of change in precorneal tear film stability, lipid layer status and tear secretion. The tear secretion were categorized into Schirmer score less than 5 mm and 5 mm or more, and precorneal tear film stability were categorized into tear film BUT less than 5 sec and 5 sec or more which is considered the definitive threshold value for dry eye [33], [34]. The tear lipid layer patterns were categorized into grade less than 3 and 3 or more. We examined the relationship between tear function and the amount of VDT use per year (<4, 4–8, 8–12, ≥12 years) and hours per day VDT (≤2, 2–4, 4–6 6–8, >8 hours). We studied linear trends by fitting categorical variables using median values for each category as continuous values in the model. The models were adjusted for age and gender.

We excluded participants who lacked data related to tear secretion (3), the amount of VDT use by year or time of VDT use per day (421). The data for the remaining 601 participants (335 males, 266 females) were used for the analyses of the relationship between amount of VDT use and tear function. Statistical analysis was performed using the JMP version 8 (SAS Institute Inc., USA).

### Rat VDT user model

#### Animals

Eight-week-old female Sprague-Dawley rats (Tokyo Laboratory Animal Science, Japan) were used for this study. At 1 week prior to the experiments, all animals were quarantined and acclimatized under the following general conditions: room temperature of 23±2°C, relative humidity of 60%±10%, an alternating 12-hour light–dark cycle (8 AM to 8 PM), and water and food available *ad libitum*. All animal experiments as follows were approved by the Animal Care and Use Committee of Ophtecs Corporation, and all procedures were performed in accordance with the Association of Research and Vision in Ophthalmology (ARVO) statement for the Use of Animals in Ophthalmic and Vision Research.

#### Simulation of VDT use

The model and methodology used for the simulation of VDT has been previously published [14], [35]. Briefly, a series of treatments were performed under dry conditions, with room temperature of 23±2°C, relative humidity of 25%±5%, and a constant air flow of 2 to 4 m/s. After being placed on a swing, each rat was kept stationary for 7.5 hours per day between 9:00 AM and 5:00 PM. Lacrimal function and morphology were evaluated after undergoing 10 days of the swing procedure, unless otherwise mentioned.

#### Assessment of restoration

In the study that examined shorter times on the swing, the amount of time the rat was on the swing was 4 and 6 hours, respectively. For the study that examined a modified resting pattern, various patterns were used during the 6-hour period the animals were on the swing. The variations used included 3 equal resting/swing periods, five 30-minute resting periods that were each followed by 1 hour on the swing, two 1-hour resting periods that were each followed by 2 hours on the swing, and one 2-hour resting period that was followed by 3 hours on the swing. Lacrimal functions were evaluated after 10 days for each of the different groups. In the study that examined stimulation of lacrimal function, tear fluid secretion was stimulated by subcutaneous injection of 0.75 mg/kg pilocarpine hydrochloride (Merck, Germany) every day after the allocated swing time. The modified Schirmer test was performed before pilocarpine injection on days 5 and 10. In the rehabilitation study, after the initial 10 days of the swing procedure, the animals were placed in cages and maintained under the same general conditions without any further time on the swing. In these animals, the recovery of the lacrimal function was evaluated on days 1, 5 and 10.

#### Measurement of tear secretion

We used a modified Schirmer test [36] on the rats' eyes to measure tear fluid secretion under topical anesthesia induced with a 0.4% oxybuprocaine hydrochloride solution (Santen Pharmaceutical Co., Ltd., Japan). After 3 minutes of anesthesia, a phenol red thread (Zone-Quick; Menicon, Japan) was placed on the temporal side of the upper eyelid margin for 1 minute. The length of the moistened area from the edge was then measured to within 1 mm.

#### Systemic pilocarpine stimulation

Changes in rat tear fluid secretion were measured under general anesthesia by intramuscular injection of an anesthesia cocktail containing ketamine and xylazine. Tear fluid secretion was stimulated by subcutaneous injection of 0.35 mg/kg to 1 mg/kg pilocarpine hydrochloride (Merck, Germany) after 15 minutes of general anesthesia. The Schirmer test was performed 5 minutes before and 30 minutes after pilocarpine injection.

#### Protein release from isolated LG

Both exorbital LGs were excised and cut into fragments of 0.5 to 1 mm with a scalpel blade. The fragments were digested with 100 U/µg collagenase (CLS III, Worthington, USA) at 37°C for 30 minutes in 10 mL oxygenated saline solution (OSS, 116 mM NaCl, 5.4 mM KCl, 1.8 mM CaCl_2_, 0.81 mM MgCl_2_, 1.01 mM NaH_2_PO_4_, 26.2 mM NaHCO_3_, 5.6 mM dextrose [pH 7.4]), and then washed three times with OSS. After pre-incubation in OSS without carbachol for 30 minutes, the LG were incubated in OSS with 10^−3^ M carbachol (Cch) (Tokyo Kasei Kougyo, Japan) or without Cch for 90 minutes. During Cch stimulation, the culture medium was exchanged every 30 minutes and maintained at 37°C during the experiment. The protein concentration in the medium was measured using the Bradford reagent (Sigma-Aldrich, USA) with bovine serum albumin used as the standard. The protein secretion rate was calculated as a percentage of the before Cch stimulation value.

#### Histopathologic examination

Animals were euthanized with an overdose of pentobarbital sodium, and their LGs were removed. For the hematoxylin and eosin staining, the LGs were fixed in 10% formalin. After dehydration, the LG specimens were embedded in paraffin, cross-sectioned, and stained. For the toluidine blue staining, the LGs were fixed with 2.5% glutaraldehyde in 0.1 M phosphate buffer (pH 7.4), then postfixed with 1% osmium tetroxide in 0.1 M phosphate buffer. After dehydration, the LGs were embedded in an epoxy resin, semithin sections were prepared and stained with toluidine blue. They were subsequently examined with a light microscope.

#### Quantification of LG cell number

Deoxyribonucleic acid content of the LG was used to calculate cell density and total cell number. The exorbital LGs were excised after the rats were given a lethal dose of sodium pentobarbital. The LG tissue was homogenized in phosphate buffered saline containing 0.5% triton X-100. Propidium iodide (100 µg/ml, Invitrogen, USA) was added to the tissue suspension and fluorescein intensity was measured at an excitation of 530 nm and emission of 620 nm. Deoxyribonucleic acid from salmon sperm (Wako Pure Chemical, Japan) was used as the standard.

#### Transmission electron microscopy

The removed LG was fixed with 2.5% glutaraldehyde in 0.1 M phosphate buffer (pH 7.4) for 1 hour. Samples were then post-fixed in 1% osmium tetroxide in 0.1 M phosphate buffer at 4°C for an hour. The LG was dehydrated in graded ethyl alcohols and embedded in Epoc 812. An ultrathin section was cut using a RT-7000 (RMC, USA), stained with uranyl acetate and lead citrate, and then examined with transmission electron microscopy (JEM-100CX; JEOL, Japan).

#### Statistical analysis

For the rat studies we used the Student's *t*-test for comparison of the two groups and the Dunnett test for multiple comparisons. The Pearson product-moment correlation coefficient was used to evaluate association between LG morphology and tear secretion. Differences between measured variables were considered significant if the resultant *P* value was 0.05 or less.

## Supporting Information

Figure S1 TEM images from intensive computer user. Lacrimal biopsy specimen photo taken by an electron microscope. Note the accumulation of abundant secretory vesicles in the acinar cells. (Scale cars: Top 5 µm; Bottom 2 µm.)
(1.82 MB TIF)

Figure S2 Recovery of LG morphology and corneal surface disorder with long-term rest without swing activity. (A) Effect of extended rest period without the swing on LG cell number After 10 days of swing use and representative H&E-stained sections of LG from recovery group. Rats were maintained 10 days under general conditions without the swing. Data represent the mean ± SEM for 8 to 16 eyes. * P<0.05 versus the normal. Scale bars  = 20 µm; (B) Effect of extended rest period without the swing on recovery of corneal surface disorder. Changes in the corneal surface disorder were studied by applying a fluorescein solution. Corneal fluorescein staining was classified with 6 levels that are based on the area of corneal staining. Data represent the mean ± SEM for 8 to 16 eyes. * P<0.05 versus the initial value. Data were analyzed by the Steel test.
(2.63 MB TIF)

Table S1 Threshold year and daily working hour for decrease in tear function.
(0.05 MB DOC)

Materials and Methods S1 (0.03 MB DOC)

Movie S1 Rat relaxing under normal conditions. The rat blinked three times during this 20 sec video clip.
(4.13 MB MOV)

Movie S2 The video was filmed under dry conditions. The frequent blinking was first observed at the point where the airflow began to face the rat.
(4.49 MB MOV)

Movie S3 The rat was placed on the swing under dry conditions with a constant airflow. No blinking was observed while the rat was on the swing.
(6.76 MB MOV)
