# Supplementary figures and images for: Lacrimal Hypofunction as a New Mechanism of Dry Eye in Visual Display Terminal Users
Source: PLoS One. 2010 Jun 15;5(6):e11119. doi: 10.1371/journal.pone.0011119 (PMC2886053; doi:10.1371/journal.pone.0011119)

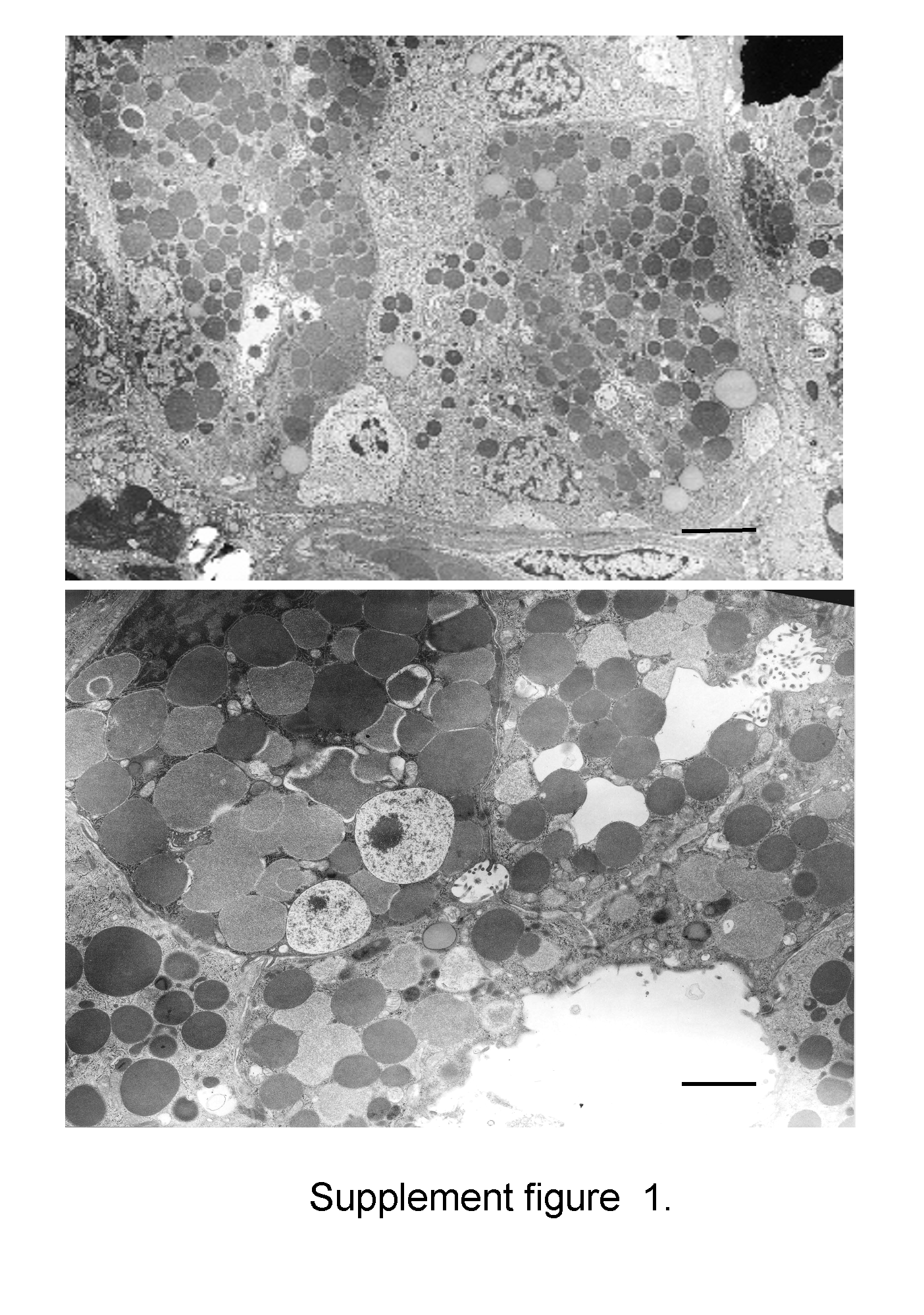

Supplement: Figure S1 — TEM images from intensive computer user. Lacrimal biopsy specimen photo taken by an electron microscope. Note the accumulation of abundant secretory vesicles in the acinar cells. (Scale cars: Top 5 µm; Bottom 2 µm.) (1.82 MB TIF) [file pone.0011119.s001.tif]

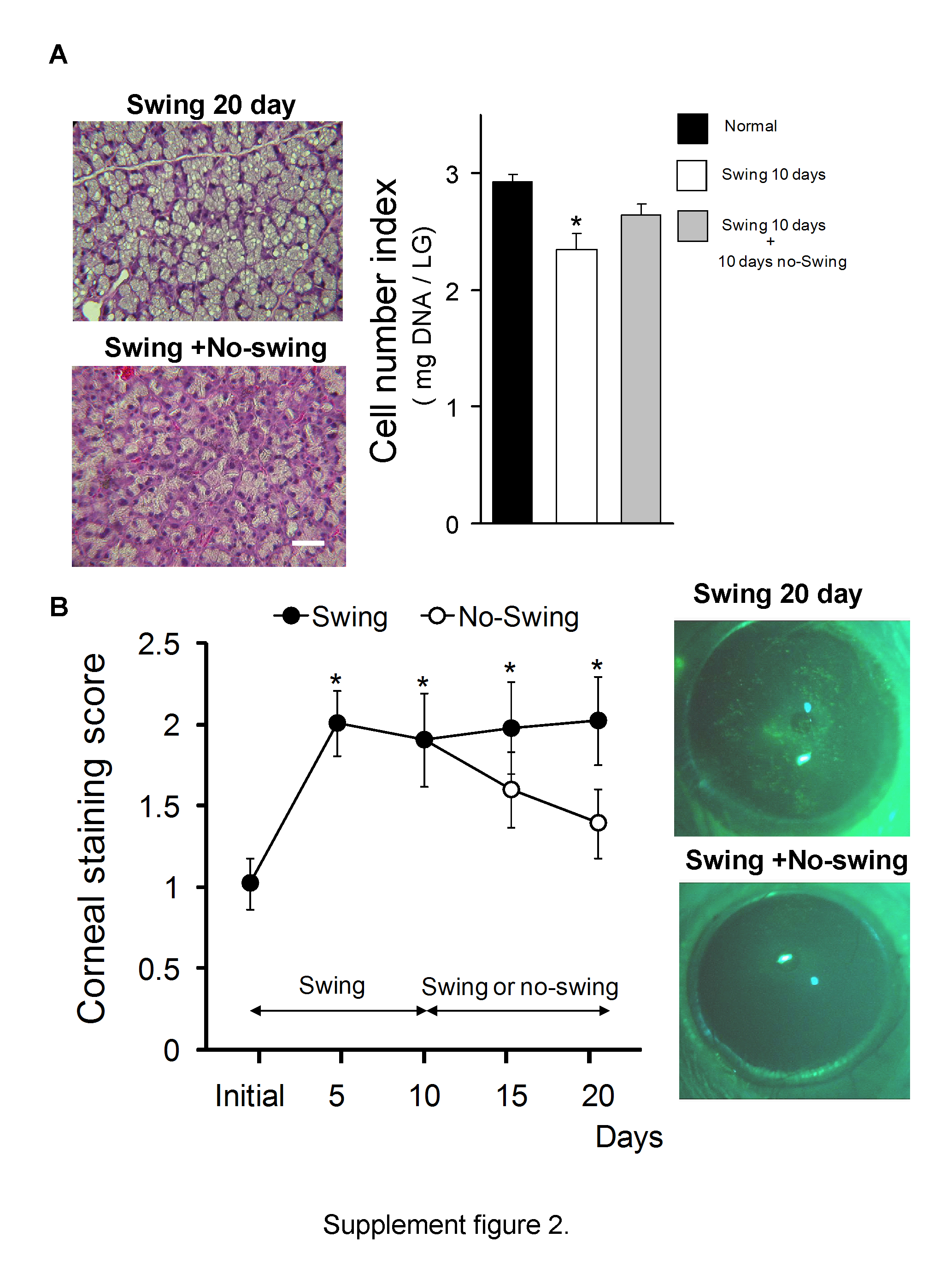

Supplement: Figure S2 — Recovery of LG morphology and corneal surface disorder with long-term rest without swing activity. (A) Effect of extended rest period without the swing on LG cell number After 10 days of swing use and representative H&E-stained sections of LG from recovery group. Rats were maintained 10 days under general conditions without the swing. Data represent the mean ± SEM for 8 to 16 eyes. * P<0.05 versus the normal. Scale bars = 20 µm; (B) Effect of extended rest period without the swing on recovery of corneal surface disorder. Changes in the corneal surface disorder were studied by applying a fluorescein solution. Corneal fluorescein staining was classified with 6 levels that are based on the area of corneal staining. Data represent the mean ± SEM for 8 to 16 eyes. * P<0.05 versus the initial value. Data were analyzed by the Steel test. (2.63 MB TIF) [file pone.0011119.s002.tif]
