# Supplementary material for: Lacrimal Hypofunction as a New Mechanism of Dry Eye in Visual Display Terminal Users
Source: PLoS One. 2010 Jun 15;5(6):e11119. doi: 10.1371/journal.pone.0011119 (PMC2886053; doi:10.1371/journal.pone.0011119)
Supplement: Table S1 — Threshold year and daily working hour for decrease in tear function. (0.05 MB DOC) [file pone.0011119.s003.doc]

Supporting information Table S1. Threshold year and daily working hour for decrease in tear function.

| **Tear function index** | **VDT working year** | |  | **Daily VDT hour** | |
| --- | --- | --- | --- | --- | --- |
|  | < 12 | ≥12 |  | < 8 | ≥ 8 |
|  | (n = 474) | (n = 127) |  | (n = 486) | (n = 115) |
| Precorneal tear stability | 5.7 ± 2.7 | 5.9 ± 2.6 |  | 5.6 ± 2.6 | 6.1 ± 3.0 |
| [BUT (sec)] |  |  |  |  |  |
| *P* value* | 0.49 | |  | 0.08 | |
| Tear lipid layer status | 2.3 ± 0.5 | 2.3 ± 0.4 |  | 2.3 ± 0.5 | 2.4 ± 0.5 |
| (DR-1　grade) |  |  |  |  |  |
| *P* value* | 0.94 | |  | 0.31 | |
| Tear secretion | 20.3 ± 10.2 | 17.2 ± 10.2 |  | 20.2 ± 9.93 | 17.5 ± 11.2 |
| [Schirmer score (mm/5min)] |  |  |  |  |  |
| *P* value* | 0.002 | |  | 0.010 | |

Values are expressed as mean ± standard deviation

*Unpaired Student's *t*-test
