# Supplementary material for: Lacrimal Hypofunction as a New Mechanism of Dry Eye in Visual Display Terminal Users
Source: PLoS One. 2010 Jun 15;5(6):e11119. doi: 10.1371/journal.pone.0011119 (PMC2886053; doi:10.1371/journal.pone.0011119)
Supplement: Materials and Methods S1 — (0.03 MB DOC) [file pone.0011119.s004.doc]

**Supporting Information**

**Materials and Methods**

***Epidemiological study in office workers engaged in VDT use***

***The classification of tear lipid layer patterns***

DR-1 images were classified from grade 1 to 5 as follows: grade 1, somewhat grey color, uniform distribution; grade 2, somewhat grey color, nonuniform distribution; grade 3, a few colors, nonuniform distribution; grade 4, many colors, nonuniform distribution; grade 5, corneal surface partially exposed.

***Lacrimal gland biopsy from the patient with VDT-related dry eye Patient***

The subject was a 42-year-old man who had performed 12 hours of VDT work daily for 15 years. He presented with corneal fluorescein staining and complained of severe ocular discomfort in both eyes. The Schirmer value was less than 5 mm, without reflex tearing, thus both upper and lower lacrimal punctum were occluded for treatment. Ocular symptoms improved after punctal occlusion treatment using punctal plugs.

***Transmission electron microscopy analysis of lacrimal gland from the patient with VDT-related dry eye***

Written informed consent was obtained in advance from the patient in accordance with the tenets of the Declaration of Helsinki. Lacrimal gland biopsy is routinely performed as part of an official examination designed to fulfill the Japanese diagnostic criteria and is approved by the ethical committee of Keio University School of Medicine. After positioning a lid retractor, a local anesthetic was injected into the superior temporal portion of the palpebral lacrimal tissue. A 1-cm incision was then made in the anesthetized region of the conjunctiva and a 5-mm-diameter specimen of LG tissue was removed and placed in fixative. No sutures were required, and digital compression was applied for 10 minutes after surgery. Specimens were immediately fixed with 2.5% glutaraldehyde in 0.1 M phosphate buffer (pH 7.4) for four hours at 4°C followed by washing three times with 0.1 M phosphate buffer.

***Rat VDT user model***

***Corneal Fluorescein Staining.***

Changes in the corneal surface were determined by applying a fluorescein solution under a blue-free barrier filter, and corneal staining of the area was graded. The total area of punctate staining was denoted as grade 0 when there was no punctate staining, grade 0.5 when less than one sixteenth was stained, grade 1 when less than one eighth was stained, grade 2 when one fourth was stained, grade 3 when greater than one half was stained, and grade 4 when the entire area was stained [13].
